# Methodology and Ontology in Microbiome Research

**DOI:** 10.1007/s13752-014-0187-6

**Published:** 2014-07-29

**Authors:** John Huss

**Affiliations:** 1 Institute of Philosophy, Jagiellonian University, ul. Grodzka 52, 31-044 Krakow, Poland; 2 Department of Philosophy, University of Akron, Akron, OH USA

**Keywords:** Enterotypes, Human Microbiome Project, Metagenomics, Philosophy of biology, Philosophy of medicine, Philosophy of science, Reification, Translational imperative, Tools-to-theories heuristic

## Abstract

Research on the human microbiome has generated a staggering amount of sequence data, revealing variation in microbial diversity at the community, species (or phylotype), and genomic levels. In order to make this complexity more manageable and easier to interpret, new units—the metagenome, core microbiome, and enterotype—have been introduced in the scientific literature. Here, I argue that analytical tools and exploratory statistical methods, coupled with a translational imperative, are the primary drivers of this new ontology. By reducing the dimensionality of variation in the human microbiome, these new units render it more tractable and easier to interpret, and hence serve an important heuristic role. Nonetheless, there are several reasons to be cautious about these new categories prematurely “hardening” into natural units: a lack of constraints on what can be sequenced metagenomically, freedom of choice in taxonomic level in defining a “core microbiome,” typological framing of some of the concepts, and possible reification of statistical constructs. Finally, lessons from the Human Genome Project have led to a translational imperative: a drive to derive results from the exploration of microbiome variation that can help to articulate the emerging paradigm of personalized genomic medicine (PGM). There is a tension between the typologizing inherent in much of this research and the personal in PGM.

The status of the human microbiome—whether it should be considered an organ, an internal feature of our developmental environment, or whether it should be assimilated into an overall ecological reconception of the human being as superorganism—is a common point of contention throughout the scientific literature (Foxman et al. 2008; Juengst 2009). Beyond adding yet another “ome” to the molecular biology lexicon, human microbiome research has generated a staggering amount of sequence data, revealing variation in microbial diversity at the community, species (or phylotype), and genomic levels (Sagoff 2012). In order to make this complexity more manageable and easier to interpret, new units—the metagenome, core microbiome, and enterotype—have been introduced in the scientific literature. In the case of the metagenome, a unit derived from the methods of bulk sequencing of environmental samples known as metagenomics, it has been proposed that the roles and identities of individual ecological actors in the community can be bypassed in favor of considering the metagenome itself as a functional unit (Committee on Metagenomics 2007). In the case of the core microbiome, an attempt has been made to find some commonality amidst the variability in microbiomes across the human subjects sampled (Turnbaugh et al. 2009). In the case of the enterotype, analyses of a relatively small and unrepresentative sample of human subjects has led to the claim that the microbiomes of all human beings can be assigned to one of three possible enterotypes (Arumugam et al. 2011; MetaHIT website).

Here, I argue that sequencing tools and exploratory statistical methods, coupled with a translational imperative, are the primary drivers of this new ontology. The phenomenon I have in mind is very much like the tools-to-theories heuristic discussed by Gigerenzer (1991, 1992): the tools of metagenomics have bequeathed us the metagenome (Juengst and Huss 2009). In addition, statistical constructs in the analysis of microbiome variation have been reified to yield enterotypes (Arumugam et al. 2011). An additional factor at work is the “translational imperative,” an attempt to reconfigure the relationship between basic research and intended clinical application (Collins et al. 2003; Maienschein et al. 2008).

These new units—metagenome, core microbiome, and enterotype—render the human microbiome more tractable and easier to interpret, and hence serve an important heuristic role. Nonetheless, there are several reasons to be cautious about these new categories prematurely “hardening” into natural units. First, virtually any environmental sample has a metagenome provided it contains DNA. With no constraint on sampling, the metagenome of an environmental sample (say, a stool sample) hardly can be said to qualify as a natural genomic or ecological unit—criteria of functional, ecological, or developmental integration are needed to assure that an actual community is being sampled. As for the core microbiome, treated as an important “hypothesis” in the literature, there is, a priori, almost certain to be some commonality in the microbiota of humans at some taxonomic level and of their microbial metagenome (microbiome) at the level of shared sequence. Standardization of taxonomic level in particular will be helpful. And the claim that there are three enterotypes into which all humans may be grouped is at best premature and at worst misleadingly essentialist. Exploratory statistical analyses of the kind used in arriving at the three enterotypes are prone to “reification” of the resulting statistical constructs (Levins and Lewontin 1980; Gould 1981). Moreover, sampling to date has been more opportunistic than representative, yielding non-robust statistical results (Jeffery et al. 2012; Koren et al. 2013). Finally, lessons from the Human Genome Project have led to a translational imperative (Collins et al. 2003; Maienschein et al. 2008): a drive to derive results from the exploration of microbiome variation that can result in new products and medical applications. Beyond showing that analytical tools are the primary drivers of the new ontology, I also suggest that translational medicine, which should be about the downstream effects of research results, also has an upstream effect on epistemology and ontology that needs to be further explored. Finally, I point out that there is a tension between the typologizing inherent in much of this research and the attention to individual variation that is alleged to be the hallmark of the emerging paradigm of personalized genomic medicine.

## What is the Microbiome?

In the scientific literature on the human microbiome, folk-etymologizing on the part of scientists has led to a curious tension. On one hand, the term “microbiome” simply refers to the collective genome of the microbiota of an organism, and is modeled after other “-omes” in molecular biology: genome, transcriptome, proteome, and the like (Lederberg 2001). This usage is relatively uncontroversial, the main points of disagreement concerning such issues as whether transient microbes or only autochthonous microbes should be included, and whether viruses should be included (or perhaps studied separately as the “virome”). On the other hand, some scientists, including several quite prominent in the field (Nicholson et al. 2005; Shade and Handelsman 2012; Weinstock 2012), have interpreted the term ecologically as a biome of microbes. Interestingly, the ecological usage seems to represent a bit of folk-etymologizing on the part of scientists, as the term microbiome (as in “microbial biome”) was scarcely used prior to the genomic era (an exception being Mohr 1952). Of course one may regard this dual usage as reflecting a dual confusion—Lederberg dubbing a new “ome” using an existing term, and others simply inferring an ecological usage in the “post-genomics” era—but overall it may be more productive to view the contrast in usage as reflecting two research strategies or points of emphasis. One strategy is fundamentally molecular, and uses the techniques of systems biology to integrate information about molecular mechanisms and pathways. The other strategy is fundamentally ecological, and uses the techniques of genomics to identify the ecological actors in the microbial community. In the biomedical literature, the ecological connotations of the term “microbiome” have been enlisted in promoting a view of the Human Microbiome Project as fostering a holistic approach to human health, even when the underlying science is genomic, and in some cases simply genetic, identifying potential functional capacities by comparison to gene databases. The polysemy of the term “microbiome” provides ecological cover for what is essentially a molecular research program.

In the United States, the Human Microbiome Project (HMP) set as its objectives the generation of a set of reference genomes for the skin, mouth, gut, lung, and vagina, including the search for a “core microbiome” at each site, the study of the relationship between microbiome variation and phenotypic variation, the development of metagenomic and bioinformatics tools, and the analysis of potential ethical, legal, and social implications of microbiome research (Turnbaugh et al. 2007). In Europe, the Metagenomics of the Human Intestinal Tract (MetaHIT) consortium has laid out a similar set of objectives but rather than studying multiple body sites has restricted its attention to the gut microbiome and its correlation with health and disease states (Ehrlich and the MetaHIT Consortium 2011).

## The Translational Imperative

Beyond the basic science of understanding which bacterial species or genes are present in and on various sites of the human body, both the Human Microbiome Project and MetaHIT have explicitly *translational* imperatives. The ultimate purpose of these projects is to understand the contribution of microbes and microbial genes to human health and wellness, in a form that *translates* into possibilities for diagnosis, intervention, wellness promotion, and disease prevention (Juengst and Huss 2009). The influence of this on research design can already be seen in the inclusion of both healthy and diseased research subjects in a study that was otherwise aimed at identifying which bacterial taxa and/or genes were shared across the guts of individual humans (Qin et al. 2010).

The term “translational imperative” was coined by Maienschein et al. (2008) to capture a shift in the relationship between basic research and an implicit social contract arising from the public funding of science. To oversimplify their nuanced historical account of this relationship, the year 2003 marked a turning point in the history of the relationship between science and its public funding in the United States. During the postwar period, funding for basic research was granted under the assumption that this research would benefit the public. By 2003, with the human genome sequenced, James Collins of the National Human Genome Research Institute (U.S.) came to recognize that a new sort of research was required that would help to actualize the medical potential of the new sequence data (Collins et al. 2003). Translational research, as this came to be called, was an attempt to incorporate consideration of the eventual application of results into the research process, to help create a pipeline from “bench to bedside.”

While Maienschein and colleagues discuss this transition and its actual and potential implications for science and policy in the U.S. context, it is clear from looking at the European initiative MetaHIT that explicit consideration of “deliverables” has informed both research design and the reporting of results. As Erlich and The MetaHIT Consortium (2011, p. 308) note:

MetaHIT seeks to be integrated in the world we live in. For this purpose, we actively participate in the international cooperation and coordination within the human metagenome field, via the International Human Microbiome Consortium (IHMC). We also seek to promote, on the one hand, the transfer of technology to industry, via an appropriate stakeholder platform, and on the other, the transfer of information about the project to the general public, by willingly accepting and even actively seeking contacts with the appropriate media.

A case in point is the identification of three distinct enterotypes into which all humans fall^1^: Enterotype 1 (dominated by *Bacteroides* sp.), Enterotype 2 (dominated by *Prevotella* sp.), and Enterotype 3 (dominated by *Ruminococcus* sp.). I will return to a discussion of enterotypes, but before doing so, it will be necessary to explore some of the methodological and ontological issues at the heart of microbiome research.

## Metagenomics

The platform technology for microbiome research has been metagenomics: “the functional and sequence-based analysis of the collective microbial genomes contained in an environmental sample” (Riesenfeld et al. 2004, p. 527). Because the vast majority of bacterial species are either difficult or impossible to culture in laboratory environments, methods of bulk sequencing the DNA of microbial consortia have been developed that bypass the need to culture the bacteria therein (Olsen et al. 1986). While Eisen has suggested that metagenomics best be regarded as a set of techniques, Dupré and O’Malley (2009) have argued that metagenomics has expanded beyond that. Either way, as I shall argue, the primary drivers of the ontology emerging from microbiome research are its tools, including metagenomics. In fact, if we follow Dupré and O’Malley in taking metagenomics to have moved beyond a set of techniques on a path toward theory, the resulting view is consistent with a common pattern in scientific change (or scientific discovery, if you like): the tools-to-theories heuristic (Gigerenzer 1991). This is when analytical tools themselves serve as a model for the theories that result from their application to data. For example, Gerd Gigerenzer has argued that cognitive psychologists using Fisherian statistical methods to analyze their experimental data have theorized that their research subjects reason like Fisherian null hypothesis testers, whereas researchers using Bayesian methods of data analysis have theorized that their subjects use Bayesian reasoning (Gigerenzer 1991, 1992). Analogously, the tool of metagenomics, which “black-boxes” species identities and arrives at a genome of an entire environmental sample has given rise to the metagenome, a community-level genome. Expressed another way, the fact that as a matter of metagenomic method, species identities do not matter suggests that in nature they do not matter either. The tools of metagenomics are giving rise to its ontology. I return to this point about tool-driven ontology in the discussion of the metagenome below.

Pace et al. (1985) are credited with devising and applying metagenomic methods, which they termed “natural population analysis” (Olsen et al. 1986). Metagenomic analysis starts with the collection of an environmental sample (e.g., from a marine environment, from soil, from acid mine drainage, or from a site on or in the human body), and the extraction of genomic DNA, which are then cut with restriction enzymes (Handelsman et al. 1998). The resulting fragments are then inserted into some sort of vector [for example, plasmids or bacterial artificial chromosomes (BACs)]. In the laboratory, bacteria (such as *E. coli*) can be induced to take up the vectors, and, when they divide, they replicate the vectors too. When all of the fragments from a given sample are represented by bacteria in this way, the resulting collection is called a library of the bacterial community. This library can then be sequenced, and the contiguous fragments aligned (sequence assembly) or else the proteins expressed by these fragments in laboratory bacteria (such as *E. coli*) can be screened for their functional capabilities (or else these can be inferred from sequence data), which is known as functional metagenomics (Committee on Metagenomics 2007). For example, Handelsman et al. (1998) discussed the screening of the soil microbiome for therapeutically useful molecules, including novel antibiotics (Handelsman 2005). The methods just described are the methods that have been classically used. Next-generation sequencing methods have substantially streamlined this process (Hall 2007; Weinstock 2012). For present purposes, the principal point is that these methods yield the metagenome of the microbial consortia present in the sample.

## Identifying Ecological Actors Using Metagenomics

Traditionally, microbial ecology has sought to identify the microbial species involved in ecological processes (Dubos et al. 1965; Savage 1977, 2001). Metagenomics has made it possible to identify a greater number of ecological actors at the level of species and higher taxonomic levels. The ability to sequence microbes without culturing them has also made it possible to develop molecular phylogenies that contain a greater number and diversity of species. The situation with bacterial phylogeny and species identification is a curious one for several reasons. For one, it has been argued that bacterial species, unlike metazoan species, are theoretical constructs, not natural units (Lawrence and Retchless 2010), or that since the genes of bacterial strains often have independent branching histories, then the bacterial species, if natural and based on evolutionary branching, must be pluralistic (Franklin 2007). Bacteria reproduce asexually, rendering the biological species concept, which relies on reproductive isolation, moot (but see Wilkins 2006 for counterargument). In addition, phylogenetically useful phenotypic traits are few and track neither species membership nor phylogeny very reliably. This has led to the idea that species membership should be defined using overall similarity at the sequence level and that certain regions of the bacterial genome could be used to infer phylogeny. Yet sequence is not only acquired vertically through common descent, but laterally through horizontal gene transfer, such that sequence similarity may result from common descent, convergence, or horizontal gene transfer.^2^

As Wilkins (2006) has pointed out, more attention has been paid to the criteria for identifying and defining bacterial species than has been paid to understanding what a species might be for these sorts of organisms. Wilkins’ point is an important one, for without it, it is easy for operational criteria, easily simplified for ease of application, to become the de facto arbiters of species membership. The 70 % rule for DNA sequence similarity and later, the 97 % rule for 16S rRNA similarity are cases in point. For reasons that need not concern us here, the Ad Hoc Committee on Reconciliation of Approaches to Bacterial Systematics in 1987 arrived at a standard for species identity based on DNA reassociation and the thermal stability of hybrids formed from DNA strands of two different bacterial strains, among other factors (Stackebrandt and Goebel 1994; Rosselló-Mora 2006). One of the criteria listed was greater than 70 % DNA–DNA similarity, which is highly correlated with thermal stabilities of the resulting hybrid, although both thermal stability and similarity were to be considered, and were expected to agree with phenotypic traits. Unfortunately, over time, many researchers have narrowed the list of criteria to a “70 % rule,” according to which 70 % DNA–DNA similarity is a sufficient criterion to assign the compared strains to the same species (Rosselló-Mora 2006). More recently, 16S rRNA has been used in species identification and phylogeny reconstruction. Since with next generation sequencing (NGS) methods, 16S rRNA comparisons are cheaper and easier than DNA hybridization studies, Stackebrandt and Goebel (1994) tested the correlation between 16S rRNA percent homology and DNA–DNA reassociation percentages. They concluded that when 16S rRNA percent homology values are less than 97 %, strains will not exceed 70 % DNA–DNA similarity and thus it can be safely inferred that they do *not* belong to the same species. Yet, in the same paper, they concluded that having a 16S rRNA percent homology *greater* than 97 % was *not* sufficient to conclude that two strains belonged to the same species. Many strains with greater than 97 % 16S rRNA sequence similarity have less than 70 % DNA–DNA similarity, which indicates that they are not conspecifics. Lamentably, over time, this rule has been misinterpreted as a positive “97 % rule” for assigning two strains to the same species (Brenner et al. 2006, p. 29). Throughout the literature, it has become commonplace for species membership to be inferred on the basis of 16S rRNA percent homology of 97 % or greater, despite the findings of Stackebrandt and Goebel (1994; for a critical examination of DNA–DNA hybridization as a phylogenetic method, the reader is referred to Rosselló-Mora 2006). The point I wish to emphasize here is the importance of operational criteria for the delineation of the species that microbiologists actually rely upon in their work. Among scientists, more effort has gone into reaching consensus on operational issues than on the biological implications of species and speciation concepts, although there is debate among some microbiologists, along with their colleagues in history and philosophy of biology, about the applicability of species and speciation concepts to bacteria (Doolittle and Papke 2006; Franklin 2006; Wilkins 2006; Doolittle and Zhaxybayeva 2010; Lawrence and Retchless 2010).

In sum, the difficulty of identifying the ecological actors in microbial ecology stems not only from the fact that most bacterial strains cannot be cultured, but also from the general problems of identifying and classifying bacteria.

## The Metagenome

Perhaps due to the difficulties in identifying and classifying bacteria, but certainly due to its offering a way around the methodological limitations of a reliance on culturing bacteria in laboratory media, metagenomics has been promoted as a way to study the functional capacities of the sampled bacterial community as a whole. For example, in *The New Science of Metagenomics* can be found such passages as:

In the end, it may be possible to view ecosystems themselves as biological units with their own genetic repertoires and to sidestep consideration of individual species. Then, both “Who is there?” and “What are they doing?” could be replaced with “What is being done by the community?” (Committee on Metagenomics 2007, p. 29).

Another part of the backdrop for bypassing species identification and explicit consideration of the ecological actors is the fact that we are losing the war on germs (Forum on Microbial Threats 2006). Microbes are developing antimicrobial resistance faster than humans can develop new antibiotics. In a paper reviewing the potential for the discovery of new antibiotics and other bioactive molecules in soil microbes, Handelsman and colleagues lay out the following strategy: “We have embarked on an effort to access the chemical diversity of soil life by cloning the metagenome of the soil without first culturing the microflora, treating the metagenome as a genomic unit” (Handelsman et al. 1998, p. R247).

The pattern of discovery to which I wish to draw attention is one that begins with the development of a novel method, in this case the ability to sequence an environmental sample without cultivating the individual organisms in the sample (Pace et al. 1985; Olsen et al. 1986; Schmidt et al. 1991). The next step is to conceive of the resulting sequence as a genomic unit, one in which the identities of the ecological actors in the community don’t matter—they can be “sidestepped.” The move here seems to be from an operational unit to a natural one. We can bypass the individuation of members, so perhaps a microbial community just *is* a set of functional potentials. In the final step, where the metagenome figures in biological explanations, it has been hardened, or reified, into a theoretical entity. We have gone from having the tools to sequence an environmental sample to thinking of the resulting sequence as a genomic unit. This is Gigerenzer’s (1991, 1992) tools-to-theories heuristic in action (Juengst and Huss 2009).

## The Core Gut Metagenome

As Sagoff (2012) has noted, the sheer quantity of sequencing data coming out of microbiome research is resulting in a data deluge. From the outset, an objective of both the Human Microbiome Project and the MetaHIT Consortium has been to develop informatics tools to analyze these data. One key question motivating microbiome research has been whether there is a core (body site-specific) microbiome common to all or most (healthy) humans (Turnbaugh 2007). Special attention has been devoted to the microbiome of the human intestinal tract (Ehrlich and the MetaHIT Consortium 2011). In 2008, microbiologist Jeremy Nicholson said the question of a core microbiome was “obsessing” microbiologists (Mullard 2008). Two points are worth noting here.

First, because biological diversity can be studied across a range of taxonomic scales from strain to kingdom, in a trivial sense there is certainly a shared set of bacterial taxa found in the guts of all sampled human subjects. The higher the taxonomic level, the greater the overall taxonomic similarity of subjects. Second, since many genes are found in the genomes of multiple bacterial taxa, it is also a virtual certainty that there are microbial genes that exist across all or most sampled human subjects (the number of shared genes actually found in a given metagenomic study is in part a function of sampling depth). The real biological interest, therefore, is in understanding the details in terms of gene function, ecological processes, physiology, and host-microbe interaction. As Shade and Handelsman (2012) put it, the study of the core microbiome should move “beyond the Venn diagram.”

A group of MetaHIT researchers, led by Junjie Qin, carried out a metagenomic analysis of the stool samples of 124 Danish and Spanish subjects, including healthy, overweight, and obese patients, and some with inflammatory bowel disease (Qin et al. 2010). One interesting result of this study was a clear separation between ulcerative colitis patients, Crohn’s disease patients, and healthy subjects when a principal components analysis was conducted on species abundance.

Finding such correlations between disease states and taxonomic composition constitutes one of the main objectives of the MetaHIT project, due to its potential for the diagnosis, treatment, or prevention of disease; but for purposes of the present paper, our main concern is the new types of genomic units that emerge from microbiome research. Qin and coworkers defined and identified two such units: a minimal gut genome and a minimal gut metagenome. The minimal gut *genome* is defined as the genes responsible for the functional capacities necessary for any bacterial species to thrive in a gut environment. Such functions include the “housekeeping” functions (e.g., main metabolic pathways) necessary for any bacterial species, as well as functions specific to life in gut environments (Qin et al. 2010). Genes for proteins involved in these functions (e.g., for adhesion to host cells) are expected to be present in all or most bacterial species in the gut. The minimal gut *metagenome* is defined as the set of genes responsible for the functional capacities necessary for the homeostasis of the whole microbial “ecosystem.” They are expected to be present in all or most subjects’ stool samples (Qin et al. 2010). In this study, both a minimal gut genome and a minimal gut metagenome were identified and described (Qin et al. 2010).

A study by Turnbaugh et al. (2009) of the gut microbiomes of lean and obese twins, found that at the level of bacterial lineages (at least among prevalent ones), there was no core gut microbiome, but that there was a core gut microbiome at the level of shared genes. As they put it:

The hypothesis that there is a core human gut microbiome, definable by a set of abundant microbial organismal lineages that we all share, may be incorrect: by adulthood, no single bacterial phylotype was detectable at an abundant frequency in the guts of all 154 sampled humans. Instead, it appears that a core gut microbiome exists at the level of shared genes, including an important component involved in various metabolic functions. This conservation suggests a high degree of redundancy in the gut microbiome and supports an ecological view of each individual as an “island” inhabited by unique collections of microbial phylotypes: as in actual islands, different species assemblages converge on shared core functions provided by distinctive components. (Turnbaugh et al. 2009, pp. 483–484)

Three points deserve emphasis here. First, in this particular study, Turnbaugh and colleagues do not attempt to differentiate between functional capabilities any bacterial species must possess (minimal gut genome) and those that must be present within the ecosystem as a whole (minimal gut metagenome). Second, in both studies, the presence of previously catalogued sequence is standing in for metabolic (ecological?) function. Function is inferred, using gene databases, from the presence of sequence. Third, both studies resisted the temptation to ascend the taxonomic scale in the search for commonality across samples. The metagenomic conception of the core gut microbiome stands in contrast to the taxonomically defined enterotypes, to which we now turn.

## Enterotypes

Researchers from the MetaHIT Consortium conducted an exploratory statistical analysis of a total of 39 fecal metagenomes from six countries and found that variation in taxonomic composition at the genus level fell into three distinct clusters that did not map neatly onto nationality or any other obvious predictors (Arumugam et al. 2011). Enterotypes 1, 2, and 3 were numerically dominated by the genera *Bacterioides*, *Prevotella*, and *Ruminococcus*, respectively. These results were published widely in venues such as *The New York Times* and *New Scientist*, and have been taken up in the scientific literature (Siezen and Kleerebezem 2011). As of this writing, “The Discovery of the 3 Enterotypes” is prominently displayed on the MetaHIT Consortium website (http://metahit.eu). Besides the sequences generated, the enterotypes are highlighted as perhaps the key finding of the project, along with their potential implications for personalized medicine.

## Are Enterotypes Robust?

The enterotype phenomenon is illuminating in what it reveals about robustness analysis. The enterotypes have withstood many tests of their robustness (Arumugam et al. 2011; Moeller et al. 2012). Once the MetaHIT researchers discovered that relative abundances of bacteria at the genus level fall into three distinct clusters, they set about to ascertain whether the enterotypes were robust (Arumugam et al. 2011). Does some other number of clusters, say two or five, capture the underlying variation equally well? Do the enterotypes hold up if some of the samples are removed? What effect does the exclusion of the most abundant genera from the data set have on the integrity of the enterotypes? Are three enterotypes still obtained if the data are randomized? Do the same enterotypes emerge from the analysis of data obtained from different subjects? After conducting analyses to answer these questions, the conclusion reached was that enterotypes are robust (Arumugam et al. 2011). Further evidence that the enterotypes are robust comes from research by Moeller et al. (2012), who replicated the MetaHIT study using fecal samples collected from a subspecies of chimpanzee in Gombe National Park. Not only were they careful to use the same statistical methods as in the MetaHIT study, but they had the additional advantage of working with longitudinal data, enabling them to test whether chimpanzee enterotypes remain stable over time (Moeller et al. 2012). They concluded that chimpanzees, too, exhibit three enterotypes, analogous to those found in the MetaHIT study, and drew the evolutionary inference that the three enterotypes predate the hominid-chimp split. Intriguingly, over the eight-year sampling period, the longitudinal data indicate that the enterotypes themselves remain stable over time, even though the chimpanzee hosts switched from one enterotype to another (Moeller et al. 2012). The enterotypes appear to exhibit an integrity—presumably ecological—that is not host-dependent.

Nonetheless, enterotypes are not as robust as they at first appeared. In particular, they are highly sensitive to taxonomic level, to the particular clustering algorithms used, and to the range of variation in the database analyzed (Koren et al. 2013). When the same data were analyzed below the genus level, enterotypes did not emerge from the analysis. Moreover, the criterion the MetaHIT researchers used to determine the number of clusters that best fit the variation in the data already presupposes that the data are significantly clustered (Arumugam et al. 2011). It does not test to see whether the variation is in fact significantly clustered (Koren et al. 2013). As a greater number of samples are added to the analysis, the variation appears to be better characterized as following a smooth gradient with samples at the end highly enriched or highly depleted in the genus *Bacteroides,* rather than a set of three discrete clusters (Jeffery et al. 2012; Koren et al. 2013).

There are two lessons here for robustness analysis. As Wimsatt (1981) emphasized in a seminal paper, one important object of robustness analysis is to search for *failures* of robustness and use them to learn about the factors that invariances depend upon. Robustness analysis is rightly classed as a heuristic, and as with any heuristic, the point is not to rely blindly on the heuristic, but to learn as much as possible from the cases in which the heuristic fails (Wimsatt 2007). More generally, robustness analysis causes fewer philosophical difficulties when it is deployed in a Popperian spirit, as an attempt to discover instances of pseudo-robustness and as one strategy for subjecting scientific findings to critical scrutiny (Popper 1959). As Koren et al. (2013) point out, “[I]f different metrics yield different results, authors should attempt to understand the discrepancies and justify their choice of distance metric.”

By studying failures of robustness in the delineation of enterotypes, more will be learned about the underlying ecological phenomena that the enterotypes are intended to capture.

## Reification

One other path to a methodologically driven ontology in the Human Microbiome Project is the path of reification. When articles appear in the microbiology and biotechnology literature with subtitles such as “Are we our enterotypes?” (Siezen and Kleerebezem 2011), it is worth pausing to consider the possibility that a process of reification is underway. To reify is to treat an abstraction as a concrete object. As Gould (1981) pointed out in the studies of *g*, the general intelligence factor, and Levins and Lewontin (1980) have discussed in their examination of the statistical practices of biologists, there is a strong temptation to treat statistical constructs as concrete objects with causal roles and a physical existence (Lewontin 1974). This doesn’t imply that all cases of reducing the dimensions of data to reveal structure are instances of reification. As Baird (1987) has made clear, one can point out that patterns in correlations in data are “really there” without committing the fallacy of reification. The point is that statistical constructs are mathematical objects and as such are tools for understanding the underlying phenomena. Statistical analyses and new bioinformatics methods are the right tools for revealing structure in messy data that is not obvious from inspection, but the resulting statistical constructs—such as enterotypes—should not be confused with the underlying biological phenomena they are intended to illuminate. This is particularly important because enterotypes have already been widely trumpeted as one of the translational products of microbiome research. Once reified in this way, the concern is that they take on a life of their own, underwriting new typologies of humankind and—for example—new probiotic products: reification enabling translation, translation begetting commercialization.

## Conclusion

As more and more microbial communities are sequenced, better bioinformatics tools are being developed, and systems biology is being deployed to synthesize and interpret the interacting components of these communities and their hosts, microbial ecology is in a state of ontological flux (Doolittle and Zhaxybayeva 2010). Philosophers of biology are debating the applicability of species concepts to bacteria (Wilkins 2006; Franklin 2007). The status of the human microbiome—whether it should be considered an organ, an internal feature of our developmental environment, or whether it should be assimilated into an overall ecological reconception of the human being as superorganism—is also up for discussion (Foxman et al. 2008; Juengst 2009). Despite this foment, progress is being made in sequencing the human microbiome and developing bioinformatics tools to analyze the avalanche of data.

A look at the scientific literature reveals that there are several factors operating simultaneously to shape an emerging ontology. What I have emphasized are the role of tools—largely metagenomic methods and statistical techniques—in shaping these ontological categories. An additional influence is the translational imperative, which shifts the influence of the potential applications of scientific research upstream in the research process, incentivizing the production of knowledge products that can be put into practice as seamlessly as possible. Reification, the fallacy of treating a statistical abstraction as a constituent of physical reality, appears to be a human tendency that can play a role in the ontological categories that are adopted, particularly in sciences like microbial ecology in which multidimensional variation requires techniques that make its patterns easier for humans to recognize, interrogate, think about, and ultimately, to use. I have suggested that robustness analysis should return to its roots as a heuristic that emphasizes the search for *failures* of robustness, and what can be learned from the specific ways in which results fail to be robust (Wimsatt 1981).

The Human Genome Project was largely completed by the time the translational imperative became a guiding light of biomedical research policy, but the prospect of personalized medicine has been important to the Human Microbiome Project from its very inception (Nicholson et al. 2005; President’s Council of Advisors on Science and Technology 2008). I have focused attention on enterotype research in part because enterotypes have been touted as holding great promise for personalized genomic medicine. In 2007, as the Human Microbiome Project was getting off the ground, Ley, Knight, and Gordon, writing in an environmental microbiology journal for a feature titled “Crystal Ball—2007,” envisioned a future in which our medical insurance cards would contain one chip for our primate genome and one for our microbiome (Ley et al. 2007). Rather than needing to encode the entire microbiome, one can certainly imagine the benefits of tailoring dietary and pharmacological regimens to the enterotype of the patient. Yet even if enterotypes, suitably refined as data are amassed and techniques improved, do hold up, there is something unsettling about a vision of personalized genomic medicine that is based upon assignment to a typological category. We should bear in mind the warning of Maienschein et al. (2008) that translational research needs to take the translation metaphor seriously, and pay close attention to the terms of the target language and who is setting those terms.

## Footnotes

1 As announced on the MetaHIT consortium main page, http://metahit.eu.

2 Even allowing horizontal gene transfer to count as a kind of sex, and erecting species on this basis, leads to its own difficulties (Franklin 2007; but see Wilkins 2006).
